# Editorial: Endocrine disruptors in obesity

**DOI:** 10.3389/fendo.2024.1526898

**Published:** 2024-12-24

**Authors:** Carmela Asteria, Paola Silvia Morpurgo, Nadia Cerutti, Julianne M. Hall

**Affiliations:** ^1^ Metabolic-Endocrine Nutrition Service, IRCCS Ospedale Galeazzi Sant’Ambrogio, Milan, Italy; ^2^ Division of Endocrinology, ASST Fatebenefratelli-Sacco, Milan, Italy; ^3^ Medicine and Dietetics Unit, ASST Pavia, Pavia, Italy; ^4^ Frank H. Netter MD School of Medicine, Quinnipiac University, North Haven, CT, United States

**Keywords:** environmental obesogens, GN: genistein, PAEs: phthalates, IL-16: interleukin-16, SUMO: small ubiquitin-like modifier, EDCs (endocrine disrupting chemicals)

Endocrine disruptors or endocrine disrupting chemicals (EDCs) are natural or man-made chemicals that can be found in various environmental pollutants and everyday products, such as pesticides, heavy metals, food packaging or pharmaceuticals. EDCs can manifest deleterious effects by interfering with multiple aspects of the endocrine system, including augmentation or antagonism of endogenous hormone action. Many EDCs are recognized as “environmental obesogens”, capable of interfering with normal endocrine regulation of metabolism, adipose tissue development and maintenance, appetite, weight, and energy balance. An expanding body of scientific evidence from animal and epidemiological studies has begun to provide links between exposure to EDCs and obesity, In addition to EDCs and dysregulated hormone signaling, this editorial also highlights new insights into the role of inflammation in obesity.

At the cellular level, obesogens can disrupt the endocrine system by altering the activity of peroxisome proliferator-activated receptors (PPARs) and steroid receptors, which are nuclear transcriptional regulators involved in lipid regulation and adipocyte differentiation. This interference can lead to changes in pro-adipogenic gene expression that ultimately contributes to adipogenesis. Genistein (GN) is a well-known bioflavonoid found in many food and plant sources. Xiang et al. highlighted how this phytoestrogen interferes with the endocrine system, modifies cellular activities, and curbs homeostasis by influencing both estrogen receptors and PPARγ, the latter of which is a pivotal switch for lipogenesis. GN acts as an obesogenic factor, inducing adipogenesis in a dose-dependent fashion, leading to lipid accumulation and transformation of stem cells into adipocytes *in vitro*.

In addition to endocrine disruptors of natural origin, phthalates (PAEs), which are mainly used as plasticizers in the manufacturing of plastic products, are established EDCs. Exposure to PAEs disrupts sex hormone levels, contributing to an increased risk of obesity. To explore the association between PAEs and obesity from the perspective of sex hormones, Zhang et al. used multiple machine learning algorithms. Notably, they found that exposure to PAEs leads to an increase in estradiol and sex hormone binding globulin (SHBG) levels, while decreasing total testosterone (TT) levels. Estradiol was positively correlated with obesity, whereas TT and SHBG exhibited negative associations. These sex hormone imbalances related to PAEs exposure may account for the propensity to obesity.

Viral infection and subsequent inflammatory reactions may be associated with obesity. Chen et al. found that brain regions are involved in the co-occurrence of Covid-19 and obesity. These regions help to maintain energy balance and homeostasis by perceiving and processing various metabolic signals in the hypothalamus. The excessive intake of saturated fatty acids can activate the innate immune system and impair adaptive immunity, leading to chronic inflammation and compromised host defense against viruses. These outcomes may be worsened by continued excessive fat intake. Moreover, COVID-19 patients with pituitary dysfunction experience changes in multiple endocrine organs, tissues, and hormone substances, making them susceptible to diabetes, obesity, and fractures. The authors suggested a close relationship between the management of pituitary diseases in the context of COVID-19 and the occurrence and development of complications.

Evidence in this issue further suggests that the cytokines released in an inflammatory state may act as obesogens. Reyes-Farias et al. suggested a potential role of IL-16 in adipogenesis, lipid and glucose homeostasis, fibrosis, and inflammation in an obesity context. Obesity is associated with low-grade inflammation mainly due to immune cell infiltration of white adipose tissue (WAT). Proinflammatory cytokines including interleukin (IL)-16 are secreted by both adipocytes and infiltrated immune cells to upregulate inflammation. IL-16 correlates with body weight, BMI, and waist circumference, and individuals with extra weight are reported to have higher IL-16 plasma levels compared with normal-weight individuals. The authors found that IL-16 could be involved in inflammation, lipid accumulation, and altered glucose signaling, contributing to the development of metabolic diseases.

Adipocytes themselves can interfere in the context of obesity. The morphology of adipocytes responds rapidly and dynamically to nutrient fluctuations. However, the adaptive hypertrophy of normal adipocytes may lead to an excessive fibrosis by inducing chronic inflammation, persistent hypoxia, and increasing myofibroblast numbers. Adipocyte-induced fibrosis not only restricts the flexible expansion and contraction of adipose tissue but also initiates the development of various diseases through cellular autocrine and paracrine effects. Zhang et al. suggested that modulation of adipocytes might provide potential therapeutic avenues for reversing pathological fibrosis in adipose tissue, achieving the anti-obesity purpose. Many browning-inducing drugs, which induce the formation of brown-like, thermogenic adipocytes in white fat depots, have been shown to have potent anti-fibrotic effects at the same time. However, at present, most of them have a wide range of targets, so the side effects should not be underestimated.

Interestingly, also alterations in the expression or activity of specific small ubiquitin-like modifier (SUMO) system components can regulate the fate of adipocytes and the function of adipose tissue. SUMOylation is a post-translational modification which plays a crucial role in the pathogenesis of metabolic disorders such as obesity, insulin resistance, and fatty liver. Xie et al. highlighted how alterations in the expression or activity of specific SUMO system components may be associated with the occurrence and development of obesity and related metabolic diseases. SUMOylation, indeed, activates brown adipose tissue (BAT) and promotes white-brown transition. Furthermore, SUMOylation may regulate the secretion of glucagon-like peptide-1 (GLP-1) and gastric hormones, contributing to the regulation of appetite and nutrient absorption in both the nervous and gastrointestinal systems.

GLP-1 receptor agonists (GLP-1 RAs) are widely used for glycemic control and weight management. However, in an extensive pharmacovigilance study, Chen et al., using the FDA Adverse Event Reporting System (FAERS) database as a global spontaneous reporting system, observed a significant association between GLP-1 RAs treatment and eight distinct categories of psychiatric adverse events. These include anxiety, stress, eating disorders, fear of injection, sleep disorders, binge eating, fear of eating, and self-induced vomiting. One of the plausible explanations for this association may be the preexisting or latent psychiatric disorder in GLP-1 RA users.

In conclusion, given the difficulty in treating obesity, it is advisable to have new research into the metabolic effects of ECD and inflammatory exposures during life. The obesogen hypothesis, together with the role of inflammation in adipogenesis, offers a window on prevention and/or intervention for this global health problem.

